# The Use of Medical Services for Low-Acuity Emergency Cases in Germany: Protocol for a Multicenter Observational Pilot Study

**DOI:** 10.2196/54002

**Published:** 2024-04-10

**Authors:** Lara Maria Nau, Gunter Laux, Attila Altiner, Joachim Szecsenyi, Rüdiger Leutgeb

**Affiliations:** 1 Department of General Practice and Health Services Research University of Heidelberg Heidelberg Germany

**Keywords:** emergency medical service, EMS, ambulance misuse, low-acuity calls, emergency department, paramedics, rescue operations

## Abstract

**Background:**

The increasing number of requests for help for acutely ill patients and their management is a major problem in the health systems of many countries, but especially in Germany. Rescue coordination centers and ambulances in Germany are increasingly overloaded. As a result, rides as a part of rescue operations have been increasing in length for years, yet a relevant proportion of these operations represent low-acuity calls (LACs). The basic objective of this pilot study is the quantitative analysis of the potential misuse of requests to the rescue control center. Indications for alternative treatment options and how to handle these treatment options in nonacute, non–life-threatening health conditions, such as minor injuries or minor infectious diseases, will be assessed. The identification of these LACs is vital in order to prevent health care resources in emergency medical care becoming inadequate.

**Objective:**

The overarching goal of this study is to determine the percentage of unnecessary rescue missions on site and subsequently to obtain an impression of the paramedics’ assessment of alternative treatment options or alternative methods of rescue transportation.

**Methods:**

This will be an exploratory, noninterventional, cross-sectional study with a quantitative approach. The study is multicentric, with 21 ambulances in 12 different locations. The data for this study were collected via a questionnaire, newly developed for this study, for rescue personnel. Additionally, secondary data from the responsible control center will be linked and processed in an initial descriptive analysis. This descriptive analysis will form the basis for a subsequent variance analysis.

**Results:**

Data collection started as projected on September 18, 2023, and was ongoing until end of November 2023. We expect the documentation of several thousand rescue operations. We expect the following study results: (1) many unnecessary rescue operations, (2) immediate on-site assessment of correct care and treatment, and (3) patients’ reasons for calling a rescue coordination center.

**Conclusions:**

To our knowledge, this is the first observational study in which acute rescue operations are recorded on site. The focus of this study is on the trained paramedics’ assessment of whether rescue operations are necessary or not. Additionally, alternative treatments, such as out-of-hours care service or primary care service, are shown for each individual case. The study also intends to cover the question of which factors are relevant and statistically significantly connected to the misuse of ambulances.

**Trial Registration:**

German Register for Clinical Studies (Deutsches Register für Klinische Studien) DRKS00032510; https://drks.de/search/en/trial/DRKS00032510

**International Registered Report Identifier (IRRID):**

DERR1-10.2196/54002

## Introduction

The number of outpatient medical treatment cases in Germany has been showing an upward trend for years [1]. Additionally, the number of rescue operations in emergency medical service (EMS) shows a continuous annual increase of about 5%, amounting to almost 16.5 million emergency ambulance and emergency missions in the 2016-2017 observation period [2-5]. This increase can also be observed in the number of rescue operations performed by the ambulance service and is similar in most European countries, as well as in the United States and Australia [6-9]. The steady increase can only partly be accounted for by demographic developments or geographical peculiarities [10,11]. Rather, a not inconsiderable proportion of EMS operations and the resulting transports to an emergency department (ED) do not seem to be indicated from a medical point of view [12-14].

In Germany, patients with minor ailments should generally be treated in medical on-call service centers out of hours when their own general practitioner (GP) is not available. GPs and specialists are obliged to work in medical on-call services; in some regions this is only out of hours, while in other regions it is 24 hours, 7 days a week. The medical on-call services are regulated by the Association of Statutory Health Insurance Physicians, that is, they are self-administered. Everybody, whether seriously or only slightly ill, can consult the ED as well as clinics and the EMS. EDs are also responsible for, preferably, emergency care but also for all other acute patient concerns. [15]. In addition to a lack of professional assessment on the part of those seeking help and ignorance of the responsibilities of the existing emergency and acute patient care structures [16,17], a variety of factors can lead to the EMS and ambulance service being alerted in nonurgent cases. Apart from the possibility of low-threshold and timely care by the ambulance service, these also include the patient-specific perception of their urgency as well as a personal need for safety [18]. Other circumstances that occasionally lead to avoidable requests for ambulance service include nursing home patients with ambulatory care–sensitive conditions, older people with many comorbidities not receiving timely outpatient consultation appointments, and people with mental health problems or somatoform disorders requiring psychological care [19-25].

The increased and undifferentiated use of the EMS means rising costs at the expense of social insurance [26] and a higher workload for emergency medical staff, who are exposed to an extremely high risk of burnout [27,28]. This also leads to overcrowded EDs and therefore to great difficulties treating patients efficiently and at a high quality outside of regular and scheduled consultations [29,30]. Besides this, the response time of the EMS tends to be more and more extended, and statutory provisions consequently cannot be observed [31,32]. Serious doubts concerning the capability of the EMS and the emergency medical system in general are being seen more and more in public debate in recent years.

German policy makers are now seriously addressing this issue after making some local short-term changes; for example, alert keywords have been restricted for ambulances on call. Recently, a German government commission proposed comprehensive modifications to the EMS. These call for extending the EMS’s entitlement to services and fees for care and treatment of minor ailments on site, not just transportation (as before). The overall aim is to reorganize and expand the existing components of the EMS [33].

The aim of this prospective cross-sectional study is the quantitative analysis of unnecessary ambulance missions, in addition to the examination of indications for alternative care options in nonacute life-threatening situations instead of transport by the ambulance service to a hospital ED. Alternative health care options should be discussed to help improve care for acute patients and reduce the misuse of ambulances.

## Methods

### Aim

This study intends to quantify the fraction of low-acuity calls (LACs) in ambulance operations, focusing on alternative treatment options. For this purpose, the trial will evaluate how often EMS personnel rate a different, usually ambulatory treatment as being more suitable because a patient has a non–life-threatening health condition that does not require any urgent medical intervention. In addition, this study aims to identify and quantify the reasons why patients without a life-threatening medical condition or other medical indications are brought to an emergency room by ambulance instead of being transferred to a more suitable location for treatment. Therefore, the 2 main questions the study plans to cover are, first, what kind of treatment patients of the EMS need, if not emergency treatment and urgent conveyance to a hospital, and second, why patients may not receive adequate medical care. A quantitative approach is required to obtain an indication of the dimensions of these individual aspects, as there is usually no standardized documentation by the control center that goes beyond the point of “patient was taken to hospital/patient was referred to GP/refusal of transport/no patient was found,” which represents only part of the actual situation. Furthermore, the study will collect and analyze data on operational statistics to determine if conditions like operation time, day of the week, accessibility to a GP, priority, and content of the alert (as set by the dispatcher in the control center) have an impact on the frequency of LAC operations. We will also evaluate socioeconomic factors, such as age and gender of the patient, as well as the impact of the operation area.

### Design

The key feature of this exploratory, noninterventional, cross-sectional study is a web-based questionnaire (Table 1) for tablets or smartphones. In the first phase, each individual question on the web-based questionnaire was reviewed by an expert panel at the Department of General Medicine at Heidelberg University Hospital, and in the second phase, the questions were discussed with the heads of operations at the rescue coordination centers, as well as with selected paramedics. The focus was on the feasibility of this pilot study. When this study is rolled out (at large scale) there also will be analyses of interrater reliability.

**Table 1:** Questionnaire for ambulance crews on emergency cases.

Item	Content
Operation number	(Free text)
Patient gender	Male Female Gender-diverse
Patient age group (years)	18-24 25-39 40-59 60-64 65-79 ≥80
Operation time on a weekend day or holiday^a^	Yes/no
Operation time during main opening hours of general practitioner care^b^	Yes/no
Classification of operation area (number of inhabitants)	Metropolitan (>100,000) Urban (30,000-100,000) Suburban (10,000-30,000) Rural (<10,000)
Working diagnosis	(Free text)
Treatment or procedure performed (multiple choices possible)	Being conveyed to a hospital Referral to a general practitioner or medical on-call service Continuance at home Patient refused being conveyed Therapeutic measures performed (Free text)
Appropriate treatment or procedure (multiple choices possible; no choice possible)	Being conveyed to a hospital autonomously Calling on the medical on-call service autonomously Consulting the general practitioner for further care Exclusively counseling Counseling by telephone possible Social caretakership Being conveyed by a patient transport ambulance (Free text)
Reasons for being conveyed to a hospital without medical indication (multiple choices possible; no choice possible)	Missed transportation opportunity by patient or relatives Patient insists on being conveyed by ambulance Social indication House call by general practitioner or medical on-call service indicated but not provided Medical on-call service not favored by patient Insecurity of team leader Language barrier (Free text)

^a^As defined by the state in which the operation took place.

^b^Main opening hours were defined as Monday, Tuesday, and Thursday from 7 AM to 7 PM, and Wednesday and Friday from 7 AM to 2 PM.

The members of the expert panel determined which crucial dimensions of medical emergency treatments will be queried, including (1) working diagnosis, which is the assessment made on site by the ambulance crew with consideration of diagnostics that can be carried out preclinically; (2) procedures, including the ambulance crew’s approach to counseling, treatment, patient transport, and referral to other caregivers; (3) adequacy of care, that is, the assessment of the most suitable form of care, which is carried out by the ambulance crew based on the situation on site; and (4) problems that arise, that is, the evaluation of possible reasons why patients are not directed to the form of care that has been assessed as being most suitable.

The training of the paramedics consists of 5 vital elements. The important questions for assessing their concerns about possible rescue operations (Textbox 1) are described in in-person lectures. The five *W* questions are integrated in the questionnaire and special training is provided.

***W* questions and example cases.**

***W* questions**

- Which typical use cases are presented?
- Who called? (Age and gender should be documented)
- Which concerns were stated? (Assessment of the emergency is an overarching goal of the study)
- When did the concerns occur? (Duration and assessment of the acute situation has to be included in the consideration)
- Where is the location? (Infrastructure, social environment, etc., should be documented)

**Examples of possible rescue operation cases integrated into the training**

- An ambulance is alerted with high priority because of high blood pressure and vertigo. On site, the crew can rule out current life-threatening conditions, only the blood pressure is slightly elevated beyond the patient’s known level after an upsetting argument a few hours before. Anamnesis brings out that the patient is familiar with vertigo and does not have any new kinds of symptoms. So, the ambulance crew cannot find any indication for conveyance at this moment and offers the patient to stay at home unless new kinds of symptoms or decline of general condition occur. The patient prefers to do so, but his daughter presses him to “have a thorough checkup in hospital” and does not concede. The patient is conveyed to the nearest hospital. After the operation, the ambulance crew fills in the questionnaire, describes their working diagnosis as “slightly elevated blood pressure without further symptoms, known vertigo without current worsening” and picks “hospital conveyance” as performed procedure, “consulting the GP in the further course” as appropriate procedure and “patient insists on conveyance by ambulance” (which includes relatives) as reason for hospital conveyance without urgent medical indication.
- An ambulance is alerted under the heading “attendance operation for fire brigade”. As there is no patient contact during the whole operation and therefore the operation is excluded from this study, the ambulance crew does not fill in the questionnaire.
- An ambulance and an emergency doctor’s car are alerted with high priority because of acute chest pain. On site, the medical examination shows evidence for an acute myocardial infarction and the patient is quickly treated and conveyed to hospital for further treatment. After terminating the operation, the ambulance crew fills in the web-based questionnaire with the items 1-9 only, because they categorize their treatment as appropriate.
- An ambulance is alerted with low priority because of a headache on a Saturday. On site, they find a young woman who is healthy except for a cold that has been lasting for “already six days” accompanied by a sinusitis and a feeling of pressure in her head. The symptoms didn’t change during the last days, and she was not satisfied by the advice of her GP and the request to await spontaneous recovery. The ambulance crew repeats the advice of the GP and recommends waiting and see and to contact the GP or alternatively the medical out-of-hours on-call service again if no improvement will occur. The patient insists on an immediate treatment, has never heard of the medical on-call service until now and claims not to be able to drive on her own, so the paramedics call the medical on-call service themselves and ask for a home call. After the operation, they fill in the web-based questionnaire, set their working diagnosis “headache associated with acute sinusitis”, pick “referral to GP or medical on-call service” and “continuance at home” as performed procedure, “consulting the GP in the further course” and “exclusively counseling” as well as “counseling telephonically possible” as appropriate procedures and select “missing transportation opportunities by patient and relatives” and add “patient didn’t know the medical on-call service” for reasons why the patient has not been directed to the appropriate caregiver straightforward.

We explained all phrases used in the questionnaire (Table 1) to precisely define the terms used. Examples for 2 terms are as follows: “social indication” means, for example, a single, immobile older person, a single mother, or a father of an ill child without his own car and without access to public transportation; “medical on-call service” means an on-call service in a suitable medical practice, self-managed by primary care physicians outside regular consultation hours or when a GP is not available.

The questionnaire will be completed once per operation by the ambulance crew, ideally in consultation with both or all 3 crew members and will represent the assessment of the most qualified crew member. The questionnaire can be filled in until the end of the operation.

The following types of operations are excluded: operations involving minor patients (aged <18 years), secondary transports or interhospital transfers, operations cancelled during the approach, operations with no patient found, provisional operations without a patient (eg, fire service operations), and operations in which the patient dies or is found dead on the incident scene. The inclusion and exclusion criteria are listed in Textbox 2.

**Inclusion and exclusion criteria for emergency case recording.**

**Inclusion criteria**

- Minimum of 1 ambulance involved in the operation (type C according to EN 1789)
- Crew members were recruited and briefed

**Exclusion criteria**

- Provisional operation without patient (eg, fire service operations)
- Secondary transport/interhospital transfer
- Cancellation during approach
- Minor patients (aged <18 years)
- Patient dies or is found dead on incident scene
- No patient found

### Setting

The study takes place in the whole area of responsibility of the integrated emergency control center of Ludwigshafen, Germany. This covers an area of about 1200 km^2^ and represents a point of call for over 620,000 inhabitants in 6 administrative districts and independent cities. Its dispatchers answer more than 210,000 calls per year, which result in more than 140,000 EMS operations (average 380 per day) and around 11,000 operations of the fire service annually. The control center has at its disposal 21 ambulances in 12 different locations, as well as 8 emergency doctor’s cars, 1 rescue helicopter, and up to 28 patient transport ambulances (figures obtained via oral communication with the management team of the integrated EMS control center of Ludwigshafen on August 29, 2023). The area of responsibility, with its 6 administrative districts and independent cities, includes the core city of Ludwigshafen as the metropolitan zone, a few cities classified as urban regions, several smaller, suburban towns, and several remote rural townships [34]. The classification of the operation areas used in this survey can be found in Table 2.

**Table 2:** Operation areas of emergency crews.

Operation area	Description
Overall	The area around Ludwigshafen, a city in the federal state of Rhineland-Pfalz, Germany
Metropolitan	The core region of Ludwigshafen
Urban	The cities Speyer, Neustadt, and Frankenthal, as well as the districts Ruchheim and Maudach of Ludwigshafen
Suburban	The cities Bad Dürkheim, Grünstadt, Schifferstadt, Haßloch, Böhl-Iggelheim, Limburgerhof, Mutterstadt, and Bobenheim-Roxheim
Rural	All townships with less than 10,000 inhabitants

### Participant Recruitment

All ambulance crew members under the responsibility of the integrated emergency control center of Ludwigshafen will have access to voluntary participation in this study. LMN, a doctoral student, will run several information sessions integrated into conferences at the individual rescue stations. Additionally, a written invitation with information will be sent to every employee via an email distribution list on the internal communications devices of the quality management system. It will include a thorough description of the study content, clarifications concerning the questionnaire, an informed consent form, and a link and QR code supplying access to the web-based questionnaire. Consent will be obtained in the web-based questionnaire; further participation is only possible after providing informed consent.

### Data Collection

During the 10-week period of data collection, access to the web-based questionnaire is available 24 hours a day, 7 days a week. The questionnaire is accessible via the link or QR code and can be filled in using a personal computer, tablet, or smartphone. The questionnaire website has been built exclusively for the study by the department conducting the study. The EMS personnel are encouraged to capture as many operations as possible during this period. The acquisition of the data sets is not bound to a certain time frame after the operations. Although the ambulance crew members must complete the questionnaire only after finishing an operation, the data sets only have to be completed within the period of data collection. The data set of each recorded ambulance operation is directly sent to a dedicated study server within the Department of General Practice and Health Services Research of the University Hospital Heidelberg via secure internet protocols. After closing of primary data collection, additional information and secondary data provided by the control center will be combined with each completely collected data set for interpretation. This comprises the time of day of the alert, the priority of the alert, the keywords of the alert, and a specific feedback code from the ambulance.

**Figure 1:**
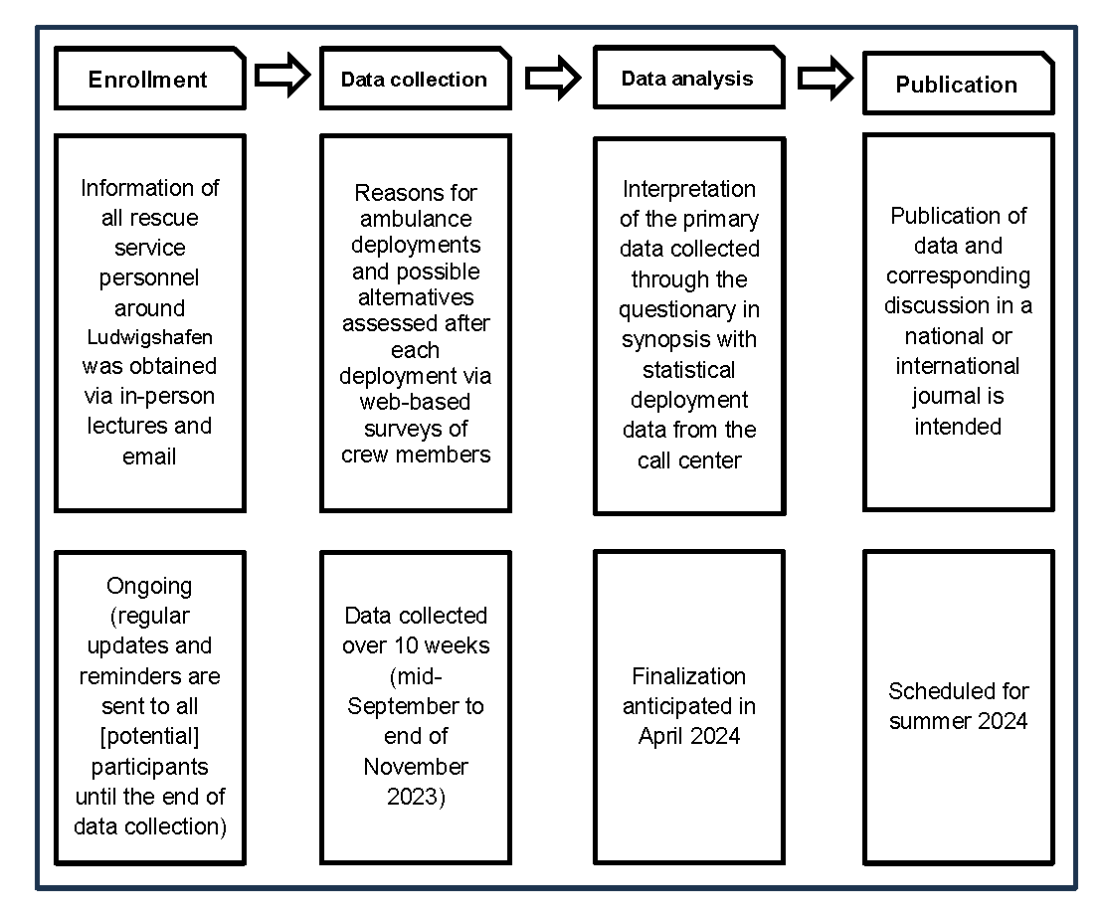
Scheduled further course of study.

The scheduled course of the study is summarized in Figure 1.

### Research Staff

The research team consists of a doctoral student, several senior researchers, and a computer scientist employed at the Department of General Practice and Health Services Research of the University Hospital Heidelberg, Germany.

### Data Management

Data management is performed by the data management team of the Department of General Practice and Health Services Research of the University Hospital Heidelberg. The secondary data are collected by the control center of Ludwigshafen and are unrelated to the described questionnaire; they are governed by their own data management team.

Each data set is directly sent to and filed on a dedicated study server in the Department of General Practice and Health Services Research of the University Hospital Heidelberg. This also applies to the required secondary data transferred from the control center. Data access is granted only to the research staff directly involved in data collection and analysis. There will be no passing on of the unprocessed data to any external institution. Additionally, secondary data from the responsible control center will be linked and processed in the initial descriptive analysis. This descriptive analysis will form the basis for the variance analysis (see Data Analysis section). Working diagnoses will be converted to *International Classification of Diseases, Tenth Edition* codes by a research physician. The free-text entries will be analyzed separately with qualitative methods to obtain a deeper insight into patients’ motives for their behavior. Data storage and extraction will be performed with MySQL Community Server x64 (Oracle Corp).

### Data Analysis

The two main outcomes for our analyses are the number and percentage of inappropriate emergency calls and the reasons for patients being conveyed to a hospital without a medical indication. This total numbers will be further subdivided according to the paramedics’ assessments. Moreover, it is of interest if there are explanatory variables such as patient gender, patient age, operation area, and operation time in the data set that might be associated with inappropriate emergency calls or being conveyed to a hospital without a medical indication. This will be analyzed based on multivariable regression modeling [35]. To calculate frequencies, rates, and percentages, we will use the PROC SQL procedure in SAS (version 9.4, SAS Institute). To perform the multivariable analyses, we will use the PROC GENMOD procedure in SAS. For qualitative analyses, we will use MAXQDA (version 24.1, VERBI Software).

### Ethical Considerations

The ethics committee of the University of Heidelberg revised and approved this study on May 30, 2023 (S-250/2023). The trial has been developed and will be executed according to all relevant national and international rules and regulations under the Declaration of Helsinki (2013 version) and International Council for Harmonisation Good Clinical Practice (E6, R2) guidelines. Consent from all participants will be obtained in the web-based form, and access to the questionnaire will be granted only after approval. Protocol modifications are not scheduled and are not foreseeable; if the need for them arises, they will have to be approved by the ethics committee. In case of modifications, a new study protocol will be released to all study participants via an official email. Due to the anonymous collection of data, participants do not have the choice to opt out after completing the questionnaire. Furthermore, as the questionnaire is anonymous, there is no possibility of inferring the identity of the participant or any other personal information. There is also no way to identify the personal data of patients using the secondary operational data provided by the control center. There is no compensation, financial or otherwise, provided to participants.

## Results

Data collection started as projected on September 18, 2023, and was ongoing until end of November 2023. We expect the documentation of several thousand rescue operations. We expect that many of these rescue operations will be unnecessary. Our intention is to train paramedics to give an immediate on-site evaluation of the correct care and treatment. We hope to identify patients’ reasons for calling a rescue coordination center so that we can give recommendations in advance on where to find the appropriate treatment options.

## Discussion

To the best of our knowledge, this is the first observational study in which acute rescue operations are being recorded on site in Germany. This study was initiated to show how many inappropriate rescue operations, such as those for minor injuries or minor infections, take place in a defined study period. The assessment of these LACs is carried out directly on site by trained rescue personnel and not later in the clinic. The assessment is therefore supplementary and can provide information on operations without the need for a referral, which is a main strength of this study. Another focus is on a preclinical assessment to determine suitable, diverse alternatives to transport by ambulance to an ED for patient conditions that do not require urgent treatment.

One major intent of our study is to complement previous work, which includes several retrospective clinical evaluations and many qualitative studies, such as interview studies, on reasons for calling the EMS in LAC situations as seen through the eyes of patients, as well as of caregivers (mostly EMS personnel), and what circumstances encourage these calls [36-38]. It is known from previous data that around 30% to 35% of all rescue service operations transporting patients to the ED are rated as inadequate and unnecessary [5-8], so a similar proportion of operations with preferred alternative care methods is expected. Most of these inadequate EMS operations involve patients whose health conditions are assessed as being able to be adequately treated by a GP or in general outpatient care [6], which is why there may be a significant difference in the number of primary care–sensitive EMS operations depending on the general availability of and access to general practices and medical out-of-hours care [17]. Additionally, we anticipate that a certain number of transportation operations by ambulance for LACs will take place because of missing transportation alternatives, which has been found to be a relevant reason for ambulance misuse in previous work [39,40]. Previous studies have shown differences in the use of emergency services between urban and rural populations [41], as well as between groups with differences in relevant socioeconomic factors, such as age and personal mobility [42], which may be confirmed in this study.

Several limitations of this study should be noted. The whole system of EMS and acute patient care is complex and includes many variables that cannot be examined in this study. For example, the control centers’ process for receiving emergency calls and dispatching ambulances plays a major role during all ambulance operations and the management of acute patients. Assessments of patients’ health condition by dispatchers, EMS personnel, and the patients themselves may greatly differ [43]. Misclassification can occur due to different assessment standards because there are usually no uniform classification criteria. There are approaches to harmonizing the triage and dispatching process, such as an intervention in out-of-hours care call centers in Germany that started in 2017 [44], but currently there exist no mandatory and coherent structures in emergency or rescue coordination centers.

In general, studies concerning the EMS in Germany deal with limited generalizability because of huge regional differences in standard operating procedures for treatment, equipment, staffing of rescue resources, and laws and regulations [45]. This is due to the federal structure in Germany and the accountability for civil protection lying mostly with the federal states or even autonomously with the local authorities [46].

Another limitation is the risk of bias. Operations that the ambulance crew rates as having been inappropriately dispatched are more likely to be captured than operations that fulfill the original functions of the EMS. Additionally, recall bias could occur if the questionnaire is not filled in soon after the end of an operation. Our intention was, in any case, to document all rescue operations regardless of urgency.

Nevertheless, the results of this cross-sectional pilot study may form the basis of further evaluations of possible interventions and pilot projects, the implementation of additional services in urgent care, support for change in the way patients are treated, and political change concerning urgent care in general.

## Abbreviations

EMS: emergency medical service
ED: emergency department
GP: general practitioner
LAC: low-acuity call

## Data Availability

Restrictions apply to the availability of the additionally processed secondary data from the emergency call center of Ludwigshafen, which were used under license for the current study, and so are not publicly available. Data are however available from the authors upon reasonable request and with permission of the emergency call center Ludwigshafen.

## References

[1] Radtke R (2022). Ambulante Behandlungsfälle in Deutschland bis 2022 [Outpatient treatment cases in Germany until 2022]. Statista.

[2] Bedarfsgerechte Steuerung der Gesundheitsversorgung 2018 [Needs-based management of health care in 2018]. Sachverständigenrat Gesundheit und Pflege [Advisory Council of Health and Care].

[3] Klein Maximilian, Schröder Hanna, Beckers Stefan K, Borgs Christina, Rossaint Rolf, Felzen Marc (2022). [Quality of documentation and treatment in the non-physician staffed ambulance: a retrospective analysis of emergency protocols from the city of Aachen]. Anaesthesiologie.

[4] Radtke R (2017). Einsatzfahrtenaufkommen in Deutschland nach Einsatzart bis 2017 [Number of rescue trips in Germany by type of operation until 2017]. Statista.

[5] Schehadat MS, Scherer G, Groneberg DA, Kaps M, Bendels MHK (2021). Outpatient care in acute and prehospital emergency medicine by emergency medical and patient transport service over a 10-year period: a retrospective study based on dispatch data from a German emergency medical dispatch centre (OFF-RESCUE). BMC Emerg Med.

[6] Andrew E, Nehme Z, Cameron P, Smith K (2020). Drivers of increasing emergency ambulance demand. Prehosp Emerg Care.

[7] Hjälte Lena, Suserud B, Herlitz J, Karlberg I (2007). Initial emergency medical dispatching and prehospital needs assessment: a prospective study of the Swedish ambulance service. Eur J Emerg Med.

[8] Patton GG, Thakore S (2013). Reducing inappropriate emergency department attendances--a review of ambulance service attendances at a regional teaching hospital in Scotland. Emerg Med J.

[9] Olshaker JS, Rathlev NK (2006). Emergency Department overcrowding and ambulance diversion: the impact and potential solutions of extended boarding of admitted patients in the emergency department. J Emerg Med.

[10] Veser A, Sieber F, Groß Stefan, Prückner Stephan (2015). The demographic impact on the demand for emergency medical services in the urban and rural regions of Bavaria, 2012-2032. Z Gesundh Wiss.

[11] Hanchate AD, Paasche-Orlow MK, Dyer KS, Baker WE, Feng C, Feldman J (2017). Geographic variation in use of ambulance transport to the emergency department. Ann Emerg Med.

[12] Metelmann B, Brinkrolf P, Kliche M, Vollmer M, Hahnenkamp K, Metelmann C (2022). [Emergency medical service, medical on-call service, or emergency department : Germans unsure whom to contact in acute medical events]. Med Klin Intensivmed Notfmed.

[13] O'Cathain A, Connell J, Long J, Coster J (2020). 'Clinically unnecessary' use of emergency and urgent care: A realist review of patients' decision making. Health Expect.

[14] Leutgeb R, Engeser P, Berger S, Szecsenyi J, Laux G (2017). Out of hours care in Germany - High utilization by adult patients with minor ailments?. BMC Fam Pract.

[15] von Stillfried D, Mangiapane S (2022). [Emergency care: need for reform from an outpatient perspective]. Inn Med (Heidelb).

[16] Agarwal S, Banerjee J, Baker R, Conroy S, Hsu R, Rashid A, Camosso-Stefinovic J, Sinfield P, Habiba M (2012). Potentially avoidable emergency department attendance: interview study of patients' reasons for attendance. Emerg Med J.

[17] Coster JE, Turner JK, Bradbury D, Cantrell A (2017). Why do people choose emergency and urgent care services? A rapid review utilizing a systematic literature search and narrative synthesis. Acad Emerg Med.

[18] Booker MJ, Simmonds RL, Purdy S (2014). Patients who call emergency ambulances for primary care problems: a qualitative study of the decision-making process. Emerg Med J.

[19] Horibata K, Takemura Y (2015). Inappropriate use of ambulance services by elderly patients with less urgent medical needs. Tohoku J Exp Med.

[20] Inoue Y, Nishi K, Mayumi T, Sasaki J (2022). Factors in avoidable emergency visits for ambulatory care-sensitive conditions among older patients receiving home care in Japan: A retrospective study. Intern Med.

[21] Leutgeb R, Berger SJ, Szecsenyi J, Laux G (2019). Potentially avoidable hospitalisations of German nursing home patients? A cross-sectional study on utilisation patterns and potential consequences for healthcare. BMJ Open.

[22] Woodhead C, Martin P, Osborn D, Barratt H, Raine R (2022). Health system influences on potentially avoidable hospital admissions by secondary mental health service use: A national ecological study. J Health Serv Res Policy.

[23] Zúñiga Franziska, Gaertner K, Weber-Schuh SK, Löw Barbara, Simon M, Müller Martin (2022). Inappropriate and potentially avoidable emergency department visits of Swiss nursing home residents and their resource use: a retrospective chart-review. BMC Geriatr.

[24] Lewis J, Weich S, O'Keeffe Colin, Stone T, Hulin J, Bell N, Doyle M, Lucock M, Mason S (2023). Use of urgent, emergency and acute care by mental health service users: A record-level cohort study. PLoS One.

[25] Voss S, Brandling J, Taylor H, Black S, Buswell M, Cheston R, Cullum S, Foster T, Kirby K, Prothero L, Purdy S, Solway C, Benger JR (2018). How do people with dementia use the ambulance service? A retrospective study in England: the HOMEWARD project. BMJ Open.

[26] (2023). Gesundheitsausgaben nach Einrichtungen (Stand 05. April 2023) [Health Expenditures going by institutions]. Statistisches Bundesamt [Federal Statistical Office].

[27] Petrino R, Riesgo L, Yilmaz B (2022). Burnout in emergency medicine professionals after 2 years of the COVID-19 pandemic: a threat to the healthcare system?. Eur J Emerg Med.

[28] Leutgeb R, Frankenhauser-Mannuß J, Scheuer M, Szecsenyi J, Goetz K (2018). Job satisfaction and stressors for working in out-of-hours care - a pilot study with general practitioners in a rural area of Germany. BMC Fam Pract.

[29] Bittencourt RJ, Stevanato ADM, Bragança Carolina Thomé N M, Gottems LBD, O'Dwyer Gisele (2020). Interventions in overcrowding of emergency departments: an overview of systematic reviews. Rev Saude Publica.

[30] Jacobson T, Salzmann M Rettungsdienste in ganz Deutschland hoffnungslos überlastet [Emergency medical services in entire Germany desperately overloaded]. World Socialist Web Site.

[31] (2022). Rettungsdienstbericht Bayern 2022 [EMS report Bavaria 2022]. Institut für Notfallmedizin und Medizinmanagement LMU Klinikum München [Institute of Emergency Medicine and Medical Management LMU Hospital Munich].

[32] (2022). Jahresbericht 2022 [Annual report 2022]. Berliner Feuerwehr [Berlin Firebrigade].

[33] Regierungskommission legt Rettungsdienst-Konzept vor - Lauterbach: Brauchen klare Strukturen [Governmental commission submits concept for EMS - Lauterbach: Have need of more distinct structures]. Federal Ministry for Health.

[34] Territorial typologies manual - cluster types. Eurostat.

[35] Grant Stuart W, Hickey Graeme L, Head Stuart J (2019). Statistical primer: multivariable regression considerations and pitfalls. Eur J Cardiothorac Surg.

[36] Kraaijvanger N, van Leeuwen H, Rijpsma D, Edwards M (2016). Motives for self-referral to the emergency department: a systematic review of the literature. BMC Health Serv Res.

[37] O'Cathain A, Simpson R, Phillips M, Knowles E (2022). Tendency to call an ambulance or attend an emergency department for minor or non-urgent problems: a vignette-based population survey in Britain. Emerg Med J.

[38] Mills B, Hill M, Miles A, Smith E, Afrifa-Yamoah Eben, Reid D, Rogers S, Sim M (2023). Calling an ambulance for non-emergency medical situations: Results of a cross-sectional online survey from an Australian nationally representative sample. Emerg Med Australas.

[39] Dejean Deirdre, Giacomini M, Welsford M, Schwartz L, Decicca P (2016). Inappropriate ambulance use: A qualitative study of paramedics' views. Healthc Policy.

[40] Little G F, Barton D (1998). Inappropriate use of the ambulance service. Eur J Emerg Med.

[41] Wong HT, Lin T, Lin J (2019). Identifying rural-urban differences in the predictors of emergency ambulance service demand and misuse. J Formos Med Assoc.

[42] Kawakami C, Ohshige K, Kubota K, Tochikubo O (2007). Influence of socioeconomic factors on medically unnecessary ambulance calls. BMC Health Serv Res.

[43] Booker MJ, Purdy S, Shaw ARG (2017). Seeking ambulance treatment for 'primary care' problems: a qualitative systematic review of patient, carer and professional perspectives. BMJ Open.

[44] Schäfer Ingmar, Menzel A, Herrmann T, Willms G, Oltrogge J, Lühmann Dagmar, Scherer M (2023). Compliance and patient satisfaction with treatment settings recommended by the medical on-call service 116117 in Germany using computer-assisted structured initial assessment: a cross-sectional observational study accompanying the demand intervention. BMJ Open.

[45] Rettungsdienstinformationen [Information on the rescue services]. Stumpf und Kossendey Verlag.

[46] Geier W Strukturen, Akteure und Zuständigkeiten des deutschen Bevölkerungsschutzes [Structures, actors and jurisdictions of the German civil protection]. Bundeszentrale für politische Bildung [German Federal Agency for Civic Education].

